# Genetic characterisation of novel, highly pathogenic avian influenza (HPAI) H5N6 viruses isolated in birds, South Korea, November 2016

**DOI:** 10.2807/1560-7917.ES.2017.22.1.30434

**Published:** 2017-01-05

**Authors:** Young-Jae Si, In Won Lee, Eun-Ha Kim, Young-Il Kim, Hyeok-Il Kwon, Su-Jin Park, Hiep Dinh Nguyen, Se Mi Kim, Jin-Jung Kwon, Won-Suk Choi, Yun Hee Beak, Min-Suk Song, Chul-Joong Kim, Richard J. Webby, Young-Ki Choi

**Affiliations:** 1 College of Medicine and Medical Research Institute, Chungbuk National University, Cheongju, Republic of Korea; 2 These authors contributed equally to this work; 3 College of Veterinary Medicine, Chungnam National University, Daejeon, Republic of Korea; 4 Department of Infectious Diseases, St. Jude Children’s Research Hospital, Memphis, Tennessee, United States

**Keywords:** Influenza A virus, H5N6, Highly pathogenic avian influenza (HPAI), South Korea

## Abstract

A novel genotype of H5N6 influenza viruses was isolated from migratory birds in South Korea during November 2016. Domestic outbreaks of this virus were associated with die-offs of wild birds near reported poultry cases in Chungbuk province, central South Korea. Genetic analysis and animal studies demonstrated that the Korean H5N6 viruses are highly pathogenic avian influenza (HPAI) viruses and that these viruses are novel reassortants of at least three different subtypes (H5N6, H4N2 and H1N1).

In late October 2016, isolation was reported of highly pathogenic avian influenza (HPAI) H5N6 virus from wild migratory birds in South Korea for the first time [1] which subsequently has caused continuous outbreaks in domestic poultry. In Southeast Asia, HPAI H5 viruses have been continuously isolated from wild birds and domestic poultry since the first detection of A/Gs/Guangdong/1/1996 (Gs/GD/1996, H5N1) in poultry in 1996 [2]. These viruses cause high mortality resulting in serious economic losses in the poultry industry and they spread widely. The HPAI H5N1 subtype was stably maintained for more than a decade before it started to evolve into the novel reassortant HPAI H5Nx virus in 2008 [3]. The HPAI H5N5, which was the first H5Nx subtype isolated, is a member of clade 2.3.4 while most H5Nx recently circulating worldwide, including H5N2, H5N6 and H5N8, cluster into a sublineage of clade 2.3.4 designated as 2.3.4.4 [4]. The clade 2.3.4.4 H5N8 influenza virus was first reported in South Korea in 2014 and subsequently spread to East Asia, Europe, and further to North America and created novel H5Nx subtypes [5-7].

In addition to the H5N8 viruses, the clade 2.3.4.4 H5N6 virus that first emerged in China in 2013, spread to Laos and Vietnam in 2014/15 with evidence of sustained transmission and further geographical spread within both countries. The H5N6 virus caused fatalities in poultry and now appears to be endemic in mainland China, Laos, and Vietnam [8]. Although the H5N8 virus is contained in clade 2.3.4.4 haemagglutinin (HA) gene pools along with HPAI H5N6 viruses, it is relatively low pathogenic in mammalian hosts [5,9], and no human cases have been reported thus far. However, the avian influenza virus subtype H5N6 caused 16 human infections including six fatalities in China as of November 2016 [10].

During late October 2016, the clade 2.3.4.4 H5N6 influenza virus was first detected in faecal specimens of migratory wild birds in South Korea and has subsequently caused poultry outbreaks in South Korea from mid-November 2016 [11]. The first reported poultry cases in Chungbuk province in central South Korea were associated with nearby die-offs of wild birds leading to speculation that migratory waterfowl were the source of infection. We report here the genetic characterisation of the H5N6 viruses isolated from faecal specimens of migratory wild birds during these first outbreaks and the investigation of their pathogenic potential in chickens.

## Genetic characterisation of novel avian influenza A(H5N6) viruses

Four H5N6 viruses were isolated from faecal samples obtained from migratory bird habitats in Chungbuk Province during a surveillance study conducted on 18 November 2016. Full-length genomic sequence analysis revealed that the viruses showed 99.9% to 100% nucleotide (nt) homology to one another (data not shown). One representative virus, A/Environment/Korea/W541/2016(H5N6), referred to as EM/W541 from here on, was selected for further study. Mitochondrial DNA sequence analysis of the faecal specimens revealed that *Anas Platyrhynchos* were the viral host [1]. Moreover, these 2016 H5N6 virus isolates belong to the A/Yunnan/0127/2015-like virus lineage (clade 2.3.4.4) detected in fatal human cases between 2014 and 2016 [12,13]. Molecular analysis demonstrated that the HA cleavage site of EM/W541 bears polybasic residues (RERRRKR/G) denoting a high-pathogenicity phenotype in chickens.

All four HPAI H5N6 virus isolates maintained the glutamine residue at position 226 (H3 numbering) and a glycine residue at position 228, which is suggestive of preferential binding to sialic acid receptors joined to sugar chains through an α-2,3 linkage, as is typical for avian influenza viruses. However, a characteristic of all H5N6 virus isolates was one amino acid deletion (133 site of HA1) relative to the other clade 2.3.4.4 HA genes (ex, MDk/Korea/W452/14), which is commonly found in avian influenza H5N6 viruses (Table).

**Table:** Molecular analysis of influenza A subtype H5 viruses emerging in November 2016 compared with previously isolated H5 viruses*

Viruses^a^	HA clade classification	HA sequence (aa)									HA deletion	NA stalk deletion	NS1			PB2 sequence at aa		Expression of PB1-F2 protein
		Cleavage site	Receptor binding sites										Deletion of aa 80–84	Aa residue at				
		335-348b	158	193	222	224	226	227	228	318	133	49-68		92	C-term	627	701	
**EM/Korea/W541/16**	2.3.4.4	RERRR_KR/G	N	N	Q	N	Q	Q	G	T	YES	YES	YES	E	ESEV	E	D	YES
Yunnan/China/0127/15^c^	2.3.4.4	RERRR_KR/G	N	N	Q	N	Q	R	G	T	YES	YES	NO	D	KPEV	K	D	YES
Ck/Sichuan/NCJPL1/2014	2.3.4.4	RERRR_KR/G	N	N	Q	N	Q	R	G	T	NO	NO	YES	E	ESEV	E	D	YES
MDk/Korea/W452/14	2.3.4.4	RERRR_KR/G	N	N	Q	N	Q	R	G	T	NO	NO	NO	D	ESEV	E	D	YES
BDk/Korea/Gochang1/14	2.3.4.4	RERRR_KR/G	N	N	Q	N	Q	R	G	T	NO	NO	NO	D	ESEV	E	D	YES
Em/Korea/W149/06	2.2	GERRRKKR/G	N	K	K	N	Q	S	G	T	NO	YES	YES	D	ESKV	K	D	YES
MDk/Korea/W401/11	2.3.2	RERRR_KR/G	D	R	K	N	Q	S	G	T	NO	YES	YES	D	ESEV	E	D	YES
Egypt/MOH/7271/14 ^c^	2.2.1.2	GERRRKKR/G	N	R	K	N	Q	S	G	T	YES	YES	YES	D	ESEV	K	D	YES

Aa: amino acid; BDk: Breeder duck; C-term: 4 amino acid sequence at the C-terminal end; Em: environment; HA: haemagglutinin; HPAI: highly pathogenic avian influenza; MDk: mallard duck; RBS: receptor binding site; MOH: Ministry of health; NA: neuraminidase; NS: nonstructural protein; PB: polymerase basic protein.

The accession numbers of each virus are followed: Yunnan/China/0127/15 : KT245143~KT245150, Ck/Sichuan/NCJPL1/2014: PB2-KM251533, PB1- KM251523, PA-KM251513, HA-KM251463, NP-KM251493, NA-KM251486, M-KM251473, and NS-KM251503, MDk/Korea/W452/14 : KJ746108~KJ746115, BDk/Korea/Gochang1/14 : KJ413831~KJ413838, EM/Korea/W149/06 : EU233731~EU233738, MDk/Korea/W401/11 : JN202558~JN202572, Egypt/MOH/7271/14 : KP702162~KP702169, and EM/Korea/W541/16 : KY272997~KY273025

^a^ The isolates in boldface are the 2016 Korean HPAI H5N6 virus examined in this study.

^b^ H3 numbering.

^c^ Human isolates.

The deletion at this position alters the 3D structure of the receptor binding unit causing an alteration of the HA receptor binding specificity and resulting in an increased affinity for the α-2,6 linkage [14,15]. A similar deletion has occurred and is maintained in 2.2.1.2 viruses in Egypt [14]. These viruses bear considerable zoonotic potential. In addition, the Korean H5N6 isolates had the characteristic 20 amino acid NA stalk deletion (49 to 68 sites) compared with the A/Ck/Sichuan/NCJPL1/2014 virus, whereas a substitution associated with resistance to NA inhibitors was not noted. The isolates also possess functional polymerase basic (PB)1-F2 proteins, which have been shown to impact on host defence mechanisms and enhance pathogenicity in vivo. However, no other mammalian-adaptive molecular determinants were observed in the viral genome [16]. The 2016 Korean virus isolates bear aspartic acid in place of glutamic acid at position 92 of the non-structural (NS)1 protein, which is responsible for attenuating anti-viral host interferon responses [17] and the C-terminal PDZ-binding motifs are both ESEV, which is typical for avian viruses and confers severe disease phenotype in mice [18].

## Phylogenetic analyses

**Figure 1:**
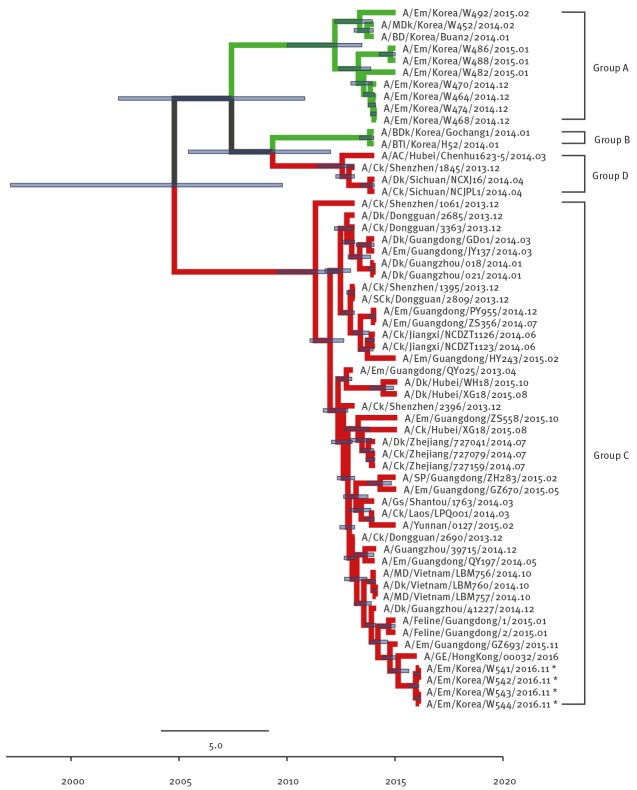
Phylogenetic tree of H5 segment of novel H5N6 viruses, South Korea, November 2016

To clarify the origins of EM/W541, phylogenetic analyses were conducted with available H5Nx virus sequences and other N6 viruses from the National Center for Biotechnology Information (NBCI) GenBank database. Phylogenetic analysis of the HA genes revealed that EM/W541 was evolutionarily close to the A/Yunnan/0127/2015-like H5N6 viruses isolated from poultry and environmental samples including fatal human cases in China during 2014–2016, and the HA genes belonged to the Group C of clade 2.3.4.4 HPAI H5 viruses (Figure 1).

AC: anas crecca; BDk: breeder duck; BD: broiler duck; BTl: baikal teal; Ck: chicken; Dk: duck; Em: environment; GE, great egret; Gs, goose; HA: haemagglutinin; MD, mallard; nt: nucleotide; SCk: silkie chicken; SP: syrrhaptes paradoxus.

To investigate the origins of novel H5N6 viruses (A/Em/Korea/W541/2016, A/Em/Korea/W542/2016, A/Em/Korea/W543/2016, and A/Em/Korea/W544/2016: marked with asterisks), full-length nt sequences of each segment were compared with available H5Nx and high blast scoring virus sequences from the GenBank. The deposited GenBank accession numbers of HA genes are KY272997-KY273000. Time-scaled phylogenies (dates shown on the horizontal axis) were inferred using strict-clock Bayesian Markov chain Monte Carlo analysis. Times of most recent common ancestors with 95% highest posterior density intervals are shown by the horizontal bars at each node (violet line). The month of isolation is indicated at the end of the viral nomenclature. The green line indicates the H5N8 subtype while the red line indicates the H5N6 subtype.

**Figure 2:**
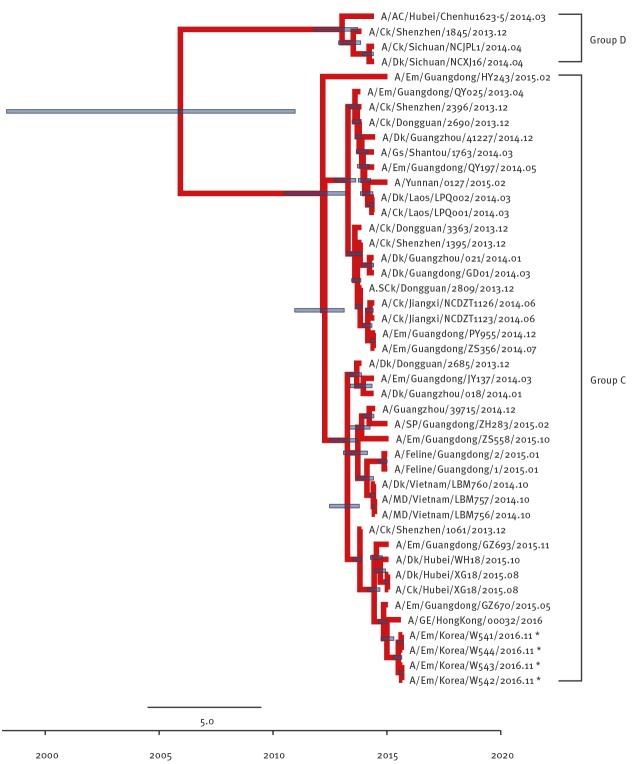
Phylogenetic tree of N6 segment of novel H5N6 viruses, South Korea, November 2016

The Group A and Group B of clade 2.3.4.4 viruses comprises H5N8 viruses identified in South Korea in 2013/14 and 2014/15 winter seasons (November to February), respectively. Group C comprises H5N6 viruses identified from China and Laos during 2013/14 and Group D comprises H5N6 viruses identified from China and Vietnam during 2013/14. The NA gene was also derived from Group C H5N6-like viruses persisting in China during 2013–2014 (Figure 2) and the most closely related strain was A/GE/Hong Kong/00032/2016.

AC: anas crecca; BDk: breeder duck; BD: broiler duck; BTl: baikal teal; Ck: chicken; Dk: duck; Em: environment; GE, great egret; Gs, goose; MD, mallard; NA: neuraminidase; nt: nucleotides; SCk: silkie chicken; SP: syrrhaptes paradoxus.

To investigate the origins of novel H5N6 viruses (A/Em/Korea/W541/2016, A/Em/Korea/W542/2016, A/Em/Korea/W543/2016, and A/Em/Korea/W544/2016: marked with asterisks), full-length nt sequences of each segment were compared with available H5Nx and high blast scoring virus sequences from the GenBank. The deposited GenBank accession numbers of NA genes are KY273005-KY273008. Time-scaled phylogenies (dates shown on the horizontal axis) were inferred using strict-clock Bayesian Markov chain Monte Carlo analysis. Times of most recent common ancestors with 95% highest posterior density intervals are shown by the horizontal bars at each node (violet line). The month of isolation is indicated at the end of the viral nomenclature. The green line indicates the H5N8 subtype while the red line indicates the H5N6 subtype.

**Figure 3:**
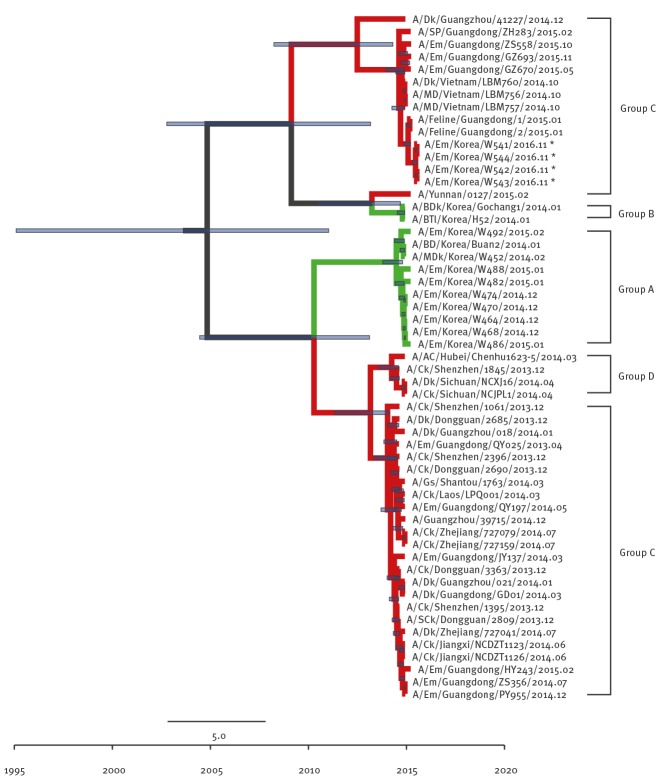
Phylogenetic tree of PB2 segment of novel H5N6 viruses, South Korea, November 2016

**Figure 4:**
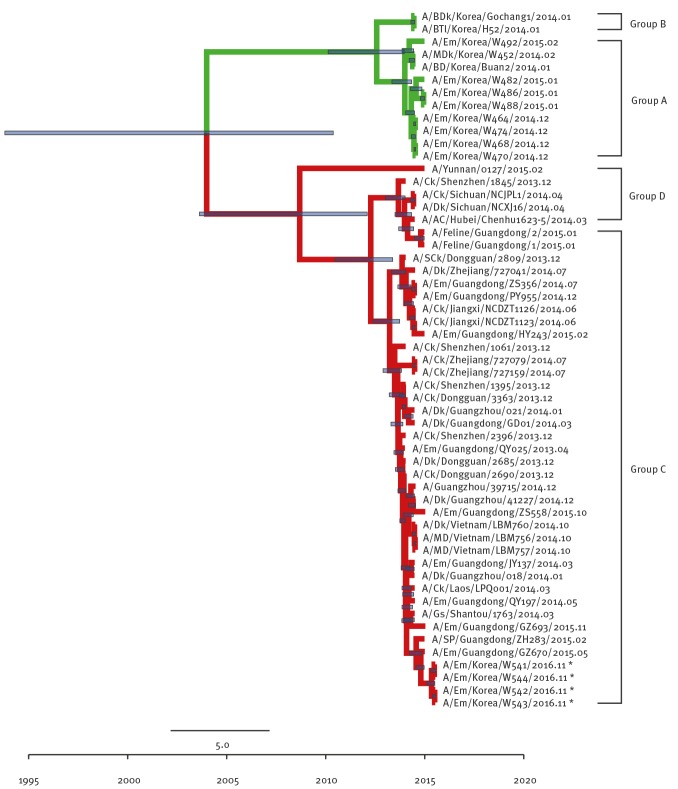
Phylogenetic tree of NP segment of novel H5N6 viruses, South Korea, November 2016

**Figure 5:**
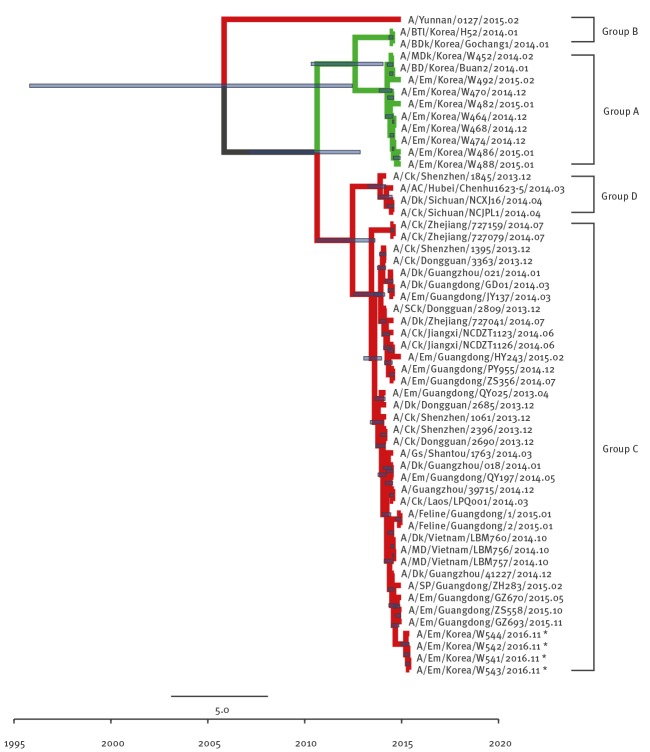
Phylogenetic tree of M segment of novel H5N6 viruses, South Korea, November 2016

**Figure 6:**
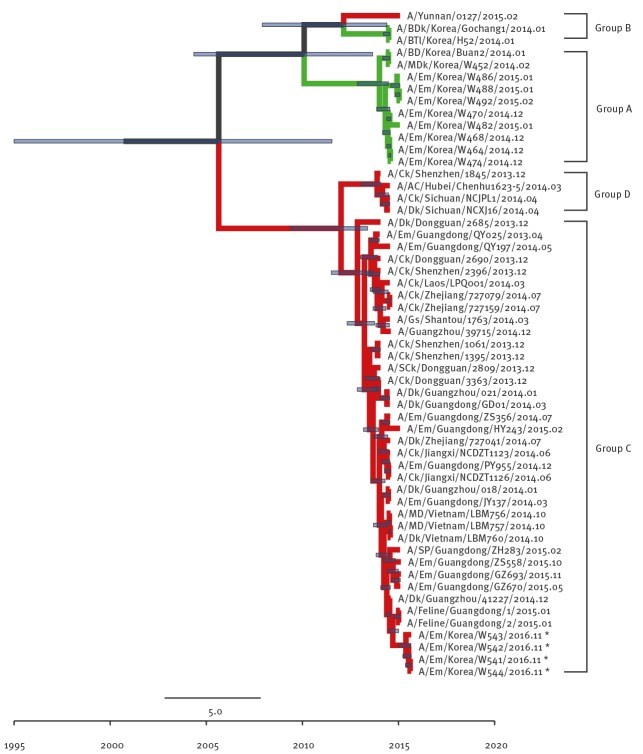
Phylogenetic tree of NS segment of novel H5N6 viruses, South Korea, November 2016

**Figure 7:**
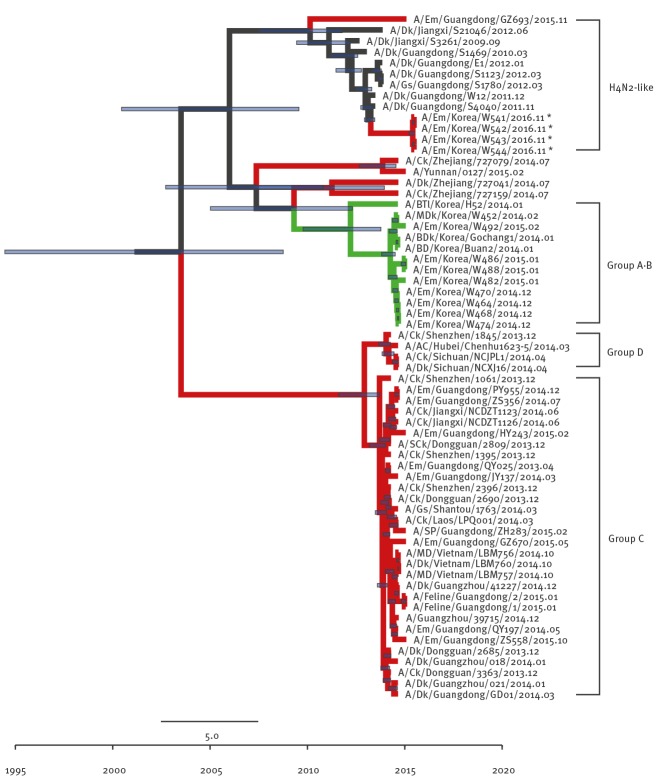
Phylogenetic tree of PB1 segment of novel H5N6 viruses, South Korea November 2016

**Figure 8:**
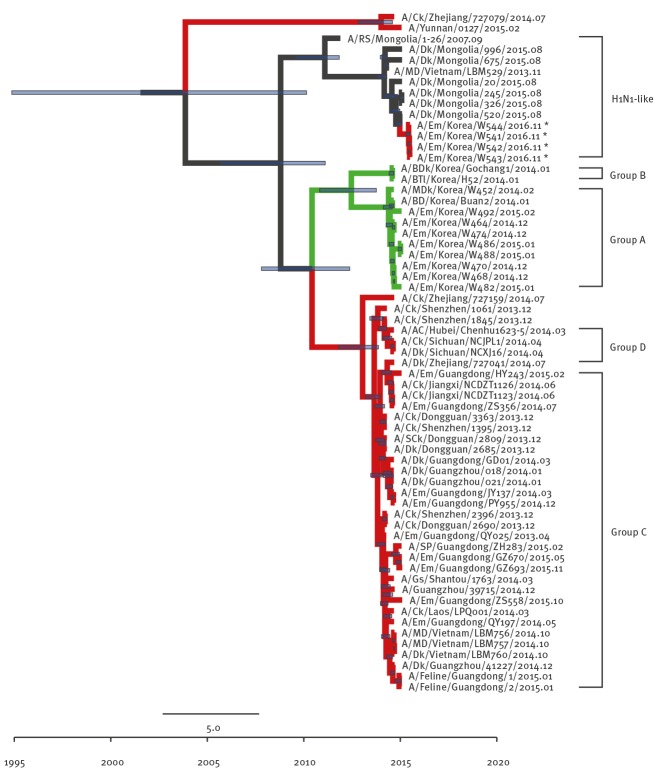
Phylogenetic tree of PA segment of novel H5N6 viruses, South Korea, November 2016

**Figure 9:**
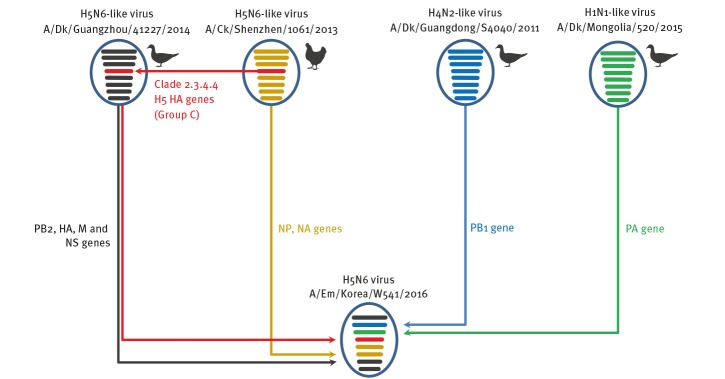
Illustration of genotypes and reassortment events resulting in the novel avain influenza H5N6 virus isolated in South Korea, November 2016

Although the other internal genes (PB2 (Figure 3), NP (Figure 4), M (Figure 5), and NS (Figure 6) can also be traced back to the Group C H5N6-like viruses(with the exception of PB1 and PA), they were clustered with different ancestors, such as A/SP/Guangdong/ZH283/2015 and A/Dk/Guanzhou/41227/2014 (H5N6)-like viruses. In contrast, the PB1 gene was closely related to A/Dk/Guangdong/S4040/2011(H4N2) strains and the PA gene was closely related to A/Dk/Mongolia/20/2015(H1N1) strains (Figures 7 and 8). The genotype map demonstrates that the first Korean H5N6 viruses were reassorted from at least three different subtypes (H5N6, H4N2 and H1N1) present within the natural gene pool in Eurasian avian influenza viruses (Figure 9).

AC: anas crecca; BDk: breeder duck; BD: broiler duck; BTl: baikal teal; Ck: chicken; Dk: duck; Em: environment; GE, great egret; Gs, goose; MD, mallard; nt: nucleotide; PB: polymerase basic protein; SCk: silkie chicken; SP: syrrhaptes paradoxus.

To investigate the origins of novel H5N6 viruses (A/Em/Korea/W541/2016, A/Em/Korea/W542/2016, A/Em/Korea/W543/2016, and A/Em/Korea/W544/2016: marked with asterisks), full-length nt sequences of each segment were compared with available H5Nx and high blast scoring virus sequences from the GenBank. The deposited GenBank accession numbers of PB2 genes are KY273025-KY273028. Time-scaled phylogenies (dates shown on the horizontal axis) were inferred using strict-clock Bayesian Markov chain Monte Carlo analysis. Times of most recent common ancestors with 95% highest posterior density intervals are shown by the horizontal bars at each node (violet line). The month of isolation is indicated at the end of the viral nomenclature. The green line indicates the H5N8 subtype while the red line indicates the H5N6 subtype.

AC: anas crecca; BDk: breeder duck; BD: broiler duck; BTl: baikal teal; Ck: chicken; Dk: duck; Em: environment; GE, great egret; Gs, goose; MD, mallard; NP: nucleoprotein; nt: nucleotide; SCk: silkie chicken; SP: syrrhaptes paradoxus.

To investigate the origins of novel H5N6 viruses (A/Em/Korea/W541/2016, A/Em/Korea/W542/2016, A/Em/Korea/W543/2016, and A/Em/Korea/W544/2016: marked with asterisks), full-length nt sequences of each segment were compared with available H5Nx and high blast scoring virus sequences from the GenBank. The deposited GenBank accession numbers of NP genes are KY273009-KY273012. Time-scaled phylogenies (dates shown on the horizontal axis) were inferred using strict-clock Bayesian Markov chain Monte Carlo analysis. Times of most recent common ancestors with 95% highest posterior density intervals are shown by the horizontal bars at each node (violet line). The month of isolation is indicated at the end of the viral nomenclature. The green line indicates the H5N8 subtype while the red line indicates the H5N6 subtype.

AC: anas crecca; BDk: breeder duck; BD: broiler duck; BTl: baikal teal; Ck: chicken; Dk: duck; Em: environment; GE, great egret; Gs, goose; M: matrix; MD, mallard; nt: nucleotide; SCk: silkie chicken; SP: syrrhaptes paradoxus.

To investigate the origins of novel H5N6 viruses (A/Em/Korea/W541/2016, A/Em/Korea/W542/2016, A/Em/Korea/W543/2016, and A/Em/Korea/W544/2016: marked with asterisks), full-length nt sequences of each segment were compared with available H5Nx and high blast scoring virus sequences from the GenBank. The deposited GenBank accession numbers of M genes are KY273001-KY273004. Time-scaled phylogenies (dates shown on the horizontal axis) were inferred using strict-clock Bayesian Markov chain Monte Carlo analysis. Times of most recent common ancestors with 95% highest posterior density intervals are shown by the horizontal bars at each node (violet line). The month of isolation is indicated at the end of the viral nomenclature. The green line indicates the H5N8 subtype while the red line indicates the H5N6 subtype.

AC: anas crecca; BDk: breeder duck; BD: broiler duck; BTl: baikal teal; Ck: chicken; Dk: duck; Em: environment; GE, great egret; Gs, goose; MD, mallard; NS: nonstructural protein; nt: nucleotide; SCk: silkie chicken; SP: syrrhaptes paradoxus.

To investigate the origins of novel H5N6 viruses (A/Em/Korea/W541/2016, A/Em/Korea/W542/2016, A/Em/Korea/W543/2016, and A/Em/Korea/W544/2016: marked with asterisks), full-length nt sequences of each segment were compared with available H5Nx and high blast scoring virus sequences from the GenBank. The deposited GenBank accession numbers of NS genes are KY273013-KY273016. Time-scaled phylogenies (dates shown on the horizontal axis) were inferred using strict-clock Bayesian Markov chain Monte Carlo analysis. Times of most recent common ancestors with 95% highest posterior density intervals are shown by the horizontal bars at each node (violet line). The month of isolation is indicated at the end of the viral nomenclature. The green line indicates the H5N8 subtype while the red line indicates the H5N6 subtype.

AC: anas crecca; BDk: breeder duck; BD: broiler duck; BTl: baikal teal; Ck: chicken; Dk: duck; Em: environment; GE, great egret; Gs, goose; MD, mallard; nt: nucleotide; PB: polymerase basic protein; SCk: silkie chicken; SP: syrrhaptes paradoxus.

To investigate the origins of novel H5N6 viruses (A/Em/Korea/W541/2016, A/Em/Korea/W542/2016, A/Em/Korea/W543/2016, and A/Em/Korea/W544/2016: marked with asterisks), full-length nt sequences of each segment were compared with available H5Nx and high blast scoring virus sequences from the GenBank. The deposited GenBank accession numbers of PB1 genes are KY273021-KY273024. Time-scaled phylogenies (dates shown on the horizontal axis) were inferred using strict-clock Bayesian Markov chain Monte Carlo analysis. Times of most recent common ancestors with 95% highest posterior density intervals are shown by the horizontal bars at each node (violet line). The month of isolation is indicated at the end of the viral nomenclature. The green line indicates the H5N8 subtype while the red line indicates the H5N6 subtype.

AC: anas crecca; BDk: breeder duck; BD: broiler duck; BTl: baikal teal; Ck: chicken; Dk: duck; Em: environment; GE, great egret; Gs, goose; M: matrix; MD, mallard; nt: nucleotide; PA: polymerase acidic; SCk: silkie chicken; SP: syrrhaptes paradoxus.

To investigate the origins of novel H5N6 viruses (A/Em/Korea/W541/2016, A/Em/Korea/W542/2016, A/Em/Korea/W543/2016, and A/Em/Korea/W544/2016: marked with asterisks), full-length nt sequences of each segment were compared with available H5Nx and high blast scoring virus sequences from the GenBank. The deposited GenBank accession numbers of PA genes are KY273017-KY273020. Time-scaled phylogenies (dates shown on the horizontal axis) were inferred using strict-clock Bayesian Markov chain Monte Carlo analysis. Times of most recent common ancestors with 95% highest posterior density intervals are shown by the horizontal bars at each node (violet line). The month of isolation is indicated at the end of the viral nomenclature. The green line indicates the H5N8 subtype while the red line indicates the H5N6 subtype.

Ck: chicken; Dk: duck; Em: environment; HA: haemagglutinin; HPAI: highly pathogenic avian influenza; M: matrix; NA: neuraminidase; NS: nonstructural; NP: nucleoprotein; PA: polymerase acidic; PB: polymerase basic.

The eight gene segments in each illustrative virus particle represent PB2, PB1, PA, HA, NP, NA, M, and NS genes (in order from top to bottom). The EM/W541 (H5N6) virus was the reassortant with at least three different subtypes (H5N6, H4N2, and H1N1) from the natural gene pool in Eurasia. Each colour represents a virus lineage (Black indicates origin from A/Dk/Guangzhou/41227/2014-like; Orange, A/Ck/Shenzhen/1061/13-like; Blue, A/Dk/Guangdong/S4040/11; Light Green, A/Dk/Mongolia/520/2015; Red, Clade 2.3.4.4 H5 HA genes).

## Virulence in chickens

To determine the pathogenicity of the EM/W541 in chickens, we initially measured the mean death times (MDT) and the intravenous pathogenicity index (IVPI) according to the recommendations outlined in the World Organisation for Animal Health (OIE) standards [19]. Briefly, 6.0 log_10_ egg infectious doses (EID_50_) /0.1 mL of the H5N6 virus were intravenously inoculated into ten 6-week-old chickens which were then monitored until death. The MDT was 36 hours and the IVPI was 2.66 in chickens, suggesting the EM/W541 virus should be classified as an HPAI virus according to OIE criteria [19].

## Conclusions

Overall, we report the identification of a novel reassortant HPAI H5N6 virus that caused large outbreaks in domestic poultry in the late 2016 winter in South Korea [11]. This H5N6 virus is a reassortant with multiple virus subtypes (H5N6, H4N2 and H1N1) from the gene pool in Eurasian avian influenza viruses. Initial animal studies revealed that this novel H5N6 virus is highly pathogenic in chickens.

At this moment, it is hard to determine whether the presented reassortment event of the H5N6 viruses occurred in 2016 during the wild bird migration into Korea or in a previous year and in another location before migration. Further detailed broad-range molecular studies are needed to elucidate when exactly the event occurred.

The first avian influenza H5N8 (clade 2.3.4.4) virus outbreak was reported in poultry in South Korea in 2014. It rapidly spread worldwide, including to Europe and North America, by migratory wild birds [20]. This rapid and wide spread underscores the need for continuous, intensive surveillance of avian influenza viruses in wild migratory birds as it can be envisaged that these viruses may be transmitted for example to Europe, or possibly worldwide, by any migratory birds that use the same migratory flyways as the birds in the previous 2014 poultry outbreak in South Korea.

## *Erratum

The cells in the first row of the Table were erroneously shifted to the left. The table was corrected and replaced on 12 January 2017.
