# Erratum for Euro Surveill. 2017;22(1)

**DOI:** 10.2807/1560-7917.ES.2017.22.2.30438

**Published:** 2017-01-12

**Authors:** Eurosurveillance editorial team

**Affiliations:** 1 European Centre for Disease Prevention and Control (ECDC), Stockholm, Sweden

In the article ‘Genetic characterisation of novel, highly pathogenic avian influenza (HPAI) H5N6 viruses isolated in birds, South Korea, November 2016’, published on 5 January 2017, the cells in the first row of the Table were erroneously shifted to the left. The table was corrected and replaced on 12 January 2017.

